# The Prevalence of Occupational Injuries and Associated Risk Factors among Workers in Bahir Dar Textile Share Company, Amhara Region, Northwest Ethiopia

**DOI:** 10.1155/2020/2875297

**Published:** 2020-07-24

**Authors:** Destaw Damtie, Abraraw Siraj

**Affiliations:** ^1^Bahir Dar University, College of Sciences, Department of Biology, Bahir Dar, Ethiopia; ^2^Ghion Preparatory School, Bahir Dar, Ethiopia

## Abstract

**Introduction:**

Occupational injuries are occurrences arising out of, or in the course of, work which results in a fatal or nonfatal injury, e.g., a fall from a height or contact with moving machines. They pose psychological, behavioral, social, vocational, and economic problems. No previous studies have been conducted on the prevalence and associated risk factors of occupational injuries among workers in Bahir Dar Textile Share Company (SC). Therefore, this study aimed to assess the prevalence and associated risk factors of occupational injury in Bahir Dar Textile SC, Northwest Ethiopia.

**Methods:**

A cross-sectional study was conducted among employees of Bahir Dar Textile SC in 2019. Three hundred (195 males and 105 females) employees were selected using proportional simple random sampling from the spinning, weaving, finishing, engineering, and administration sections.

**Results:**

The one-year and the two-week occupational injury prevalences were 42.7% and 6.7%, respectively. The one-year report from all the respondents shows that abrasion (10.7%) and eye injury (7.7%) were the two top injuries, hands (12.7%) and eyes (7.7%) were the top injured body parts, and machines (22.7%) and falling/slipping (6.3%) caused most injuries. Statistically significant differences in injuries (*p* < 0.05) were observed due to variations in gender, job category, exposure to vibration, exposure to rays/welding sparks, and labor-intensive work. The weaving section was positively associated with occupational injuries at AOR = 4.497 and *p*=0.05.

**Conclusions:**

Occupational injuries prevalence is high over the last year. The major causes of injuries were machines and falling/slipping, while the major injuries were abrasions, eye injuries, sprains, and burns. The factors significantly associated with occupational injuries were male gender, job category, use of vibrating tools, high intensive work, and rays/welding sparks. Occupational safety and health training, use of personal protective equipment (PPE), and shifting employees from intensive works are recommended.

## 1. Introduction

Ethiopia is applying the second growth and transformation plan (GTP II) to bring economic transformation and accelerated growth towards the realization of the national vision to become a low middle-income country by 2025. The GTP II aimed to raise the textile and garment industry by 80% by the end of the plan period (2019/20). Accordingly, it is planned to manufacture USD 2.18 billion and earn USD 779 million in revenue from this sector. The sector will create 174,000 job opportunities and reduce carbon emission by 25% [1].

Occupational injuries are epidemic problems in the field of public health in developing countries [2]. This is mainly because the focus on occupational health and safety, including the prevention of occupational injuries, is very limited in low-income countries [3]. For example, in a review of articles published from low-income countries especially from the East African area, the total rate of significant injuries is estimated at 40,000 per 100,000 workers per year [4]. Millions of injury cases occur every year globally [5]. In 2014, the numbers of global fatal and nonfatal occupational accidents were 381 thousand and 374 million, respectively [6]. In Ethiopia, the one-year prevalence of occupational injuries was 31.4% among workers in the Arba Minch textile factory [7], 36.9% among workers of Kombolcha textile factory [8], and 40.8% among workers of Ayka Addis textile factory [9].

These occupational injuries were associated with different risk factors: age (<30 years), male gender, health and safety training, sleeping disturbance, and job stress [10]; extra hour duty, health and safety training, workplace supervision, personal protective equipment use, and job stress in Arba Minch textile factory [7]; sex, service year and ventilation of working unit in textile factory workers in Northwest Ethiopia [11]; PPE use and sleeping disorder among workers of Ayka Addis textile factory [9]; working >48 hrs/wk, handling objects, visual concentration, timely maintenance of the machine, and sleep disorder in Kombolcha textile factory [12].

Occupational injuries pose psychological, behavioral, social, vocational, and economic problems on the employee, employer, and society [3, 13, 14]. According to the 2017 International Labor Organization (ILO) estimates, the global and African gross domestic product (GDP) losses due to occupational accidents and work-related diseases were 3.94% and 4%, respectively [15].

The authors of the present study, thus, were interested to examine the one-year [10] occupational injuries and associated risk factors among workers in Bahir Dar Textile SC. Bahir Dar Textile SC was founded in 1961 by the Italian government grant to Ethiopia as a war compensation [16]. It is the largest factory in the Amhara region and close to Bahir Dar University. It creates job opportunities for a lot of employees and has a big share in the country's economy. Since 2013, the company gave priority to the international market and exports its products to western countries. It manufactures yarn produced in the open-end and ring line, quilt cover, pillowcase, flat sheet, gray fabric, died/printed sheets, and other home textile products [17].

In Ethiopia, industrial production is growing (10.5%) and incorporates 7.4% of the labor force and contributes to 27.26% of the GDP [18]. Occupational injuries, thus, may affect the labor force and the country's economy as well as GTP II goals [1, 19]. No studies on occupational injuries and their associated factors have been conducted in Bahir Dar Textile SC. Furthermore, findings from studies in this factory can apply to other factories in Ethiopia. Therefore, the present study aimed to assess the one-year and two-week [7] prevalence and associated risk factors of occupational injury in Bahir Dar Textile SC, Northwest Ethiopia. It will provide information to the company to take injury prevention measures; to the country to make appropriate policies; to the workers to take caution.

## 2. Materials and Methods

### 2.1. Study Design

A cross-sectional study was conducted among employees of Bahir Dar Textile SC, Northwest Ethiopia, in 2019.

### 2.2. Study Setting

The study was conducted in Bahir Dar Textile SC. Bahir Dar Textile SC is found in Bahir Dar City, Northwest Ethiopia. It has 1373 workers (773 males and 600 females). All workers from spinning (224), weaving (245), finishing (292), engineering (296), and administration (316) sections were included as the study population. The shareholders of the company are the Ethiopian government and the Japanese group with a share of 65/35% shareholding, respectively [20]. The company utilizes organic cotton as the only raw material. The spinning section has the capacity of producing 15 tons of yarn per day. Weaving section has the capacity of producing 50-thousand-meter fabric, and the processing and finishing section can produce 82 thousand m^2^ of fabric per day. The garment section can produce 10 thousand pairs of bed sheets per day [17].

### 2.3. Participants

The source population was all workers from the four sections of the factory. Of these, those who had worked for at least one year [7, 10] before the study period and who were selected by proportional simple random sampling technique were included, while workers who were on sick, annual, maternity, and family leaves during the data collection and those who refused to give consent were excluded from the study.

### 2.4. Sampling Technique and Procedure

The sample size was calculated using a standard formula for a known population [21]. A 95% probability of obtaining the population proportion of workers within a 5% margin of error was assumed to be 50% as there was no data on the prevalence of occupational injuries and associated factors among workers in Bahir Dar Textile Share Company. The number of employees allocated in each section was based on the total number of employees in each section divided by the total number of employees of the company. The results were multiplied by the sample size. Finally, a total sample size of 300 (195 males and 105 females) were selected using proportional simple random sampling techniques by using the register as the sample frame.

### 2.5. Data Collection and Data Quality Control

Data were collected using a semistructured interviewer-administered questionnaire from 300 workers selected using proportional simple random sampling techniques. The questionnaires were prepared in English and translated to Amharic and back to the English language by independent experts to retain its consistency. The main contents of the questionnaire were sociodemographic characteristics (gender, age, religion, ethnicity, marital status, educational level, residence, job category, employment status, work experience, employment status, work experience, and monthly salary); environmental factors (noise, vibration, rays/welding sparks, workplace light, workplace temperature, chemical hazards, dusty work area, biological hazards, working by standing, labor-intensive work, and work supervision); behavioral factors (alcohol consumption, khat chewing, cigarette smoking, sleeping disturbance, job satisfaction, and PPE use). The data were collected by two nurses and two supervisors after two days of training following the training manual developed beforehand.

Before the actual data collection was conducted, a pretest was done in 5% (25 workers) in Bahir Dar traditional weaver workers, and inputs from the pretest were used to modify the questionnaire more suitably to generate the desired data. The interview was conducted in a confidential setting and the interviewers were supervised at each site, and regular meetings were held between the data collectors, supervisors, and principal investigator. Moreover, consistency was checked before, during, and after entering the data into the computer.

### 2.6. Operational Definitions

- *Occupational Injuries*. They are personal injuries resulting from occupational accidents sustained on workers of Bahir Dar Textile SC in connection with the performance of their works in the past year [22].
- *Personal Protective Equipment (PPE)*. It includes goggles, helmet, face shield, gloves, boots, and specialized clothing that is designed to protect parts of the body including the eyes, face, hands, figure, and feet [23].
- *Job Satisfaction*. It is any combination of psychological, physiological, and environmental circumstances that cause a person to truthfully say “I am satisfied with my job” [24].
- *Safety Training*. OSH training is given to Bahir Dar Textile SC workers.
- *Workplace Supervision*. Regular supervisions are done by OSH responsible bodies in the working sections of the factory.
- *Working Section*. It is one of the manufacturing units in Bahir Dar Textile SC.
- *Khat Chewing*. It is the practice of chewing khat leaves by the worker at least once per week.
- *Cigarette Smoking*. It is the regular inhalation of the gases and hydrocarbon vapors generated by slowly burning of cigarettes.
- *Sleeping Disturbance*. It is the presence of sleeping problems when the worker is at work in the factory.
- *Chemical Hazards*. They include fumes, dust, and gases, especially from dyes.
- *Biological Hazards*. They are organisms or organic substances that are harmful to the health of workers in Bahir Dar Textile SC. They include pests/parasites, viruses, bacteria, fungi/mold, and protein [25].
- *Labor-Intensive Work*. This refers to a process or industry that requires a large amount of labor to produce its goods or services [26].
- *Occupational Noise*. Noise “so loud it required one to speak in a raised voice to be heard” and “at arm's length” as this vocal effort suggests noise exposures exceeding 85 to 90  dBA [27].

### 2.7. Statistical Analysis

Statistical analysis was performed using statistical package for social sciences (SPSS) software version 23. Descriptive statistical methods, frequencies, and percentages were computed to summarize sociodemographic characteristics and injury prevalence of the workers. Bivariate and multivariate regressions were applied to explore the association between occupational injuries and associated risk factors at a 95% confidence interval (95% CI). Only variables with *p* values ≤0.25 in the bivariate analysis were analyzed using multivariate logistic regression analysis.

### 2.8. Ethical Considerations

The study was conducted after appropriate ethical clearance was obtained from the Research Ethics Committee of the Science college of Bahir Dar University on 10^th^ April 2019. Informed written consent was also taken from each participant.

## 3. Results

### 3.1. Sociodemographic Characteristics

A total of 300 respondents (response rate = 100%) participated in the study. One hundred ninety-five (65.0%) of the participants were males. The participants aged 22–29 years old accounted for 69.3% of the study population, 293 (97.7%) were Orthodox Christians, 297 (99.0%) were ethnically Amharas, and 159 (53.0%) were married. Two hundred twenty (73.3%) participants had a higher education, 296 (98.7%) were urban dwellers, 69 (23.0%) were administrative staff, 296 (98.7%) were permanent employees, 108 (36%) earned monthly salary ranging from 2458 to 3941 Birr (72.81 to 116.74 USD), and 158 (52.7%) had a working experience of less than five years (Table 1).

### 3.2. Prevalence of Occupational Injuries

The one-year and two-week prevalence of occupational injury were 42.7% (128) and 6.7% (20), respectively. The most prevalent annual injuries were abrasion (32; 10.7%) and eye injury (23; 7.7%), and hands (38; 12.7%) and eyes (23; 7.7%) were the major body parts injured, and the main causes of injury were machines (68; 22.2%) and falling/slipping (19; 6.3%) (Table 2). Lack of PPEs (59; 19.7%) and lack of safety training (18; 6%) were the two top reasons for injury. Eighty-four (30%) victims were hospitalized, and 926 working days were lost as a result of the 128 occupational injuries. Most injuries occurred on Monday and Friday, 14 (4.7%) and 12 (4.0%), respectively. Many of the injuries (31; 10.3%) occurred in the afternoon (1:00 PM–6:00 PM) followed by those in the morning (28; 9.3%) (7:00 AM–12:00 AM).

### 3.3. Association of the Prevalence of Occupational Injuries with Risk Factors

Results from the bivariate logistic regression (crude odds ratio = COR) (Table 3) show that female workers were less likely to be injured compared with males (COR = 0.421, *p*=0.001). The administrative staff were the least (COR = 0.436, *p*=0.039) and workers from the weaving section were the highest (COR = 4.556, *p*=0.001) injured.

Results from the multiple logistic regression (adjusted odds ratio = AOR) also showed that female workers were less likely to face occupational injury compared with male workers (AOR = 0.318, *p*=0.001). Weaving section workers had 4.497 times higher odds of occupational injury compared with workers of the spinning section (AOR = 4.497, *p*=0.005). Workers who were not exposed to vibration were less likely to face occupational injury compared with workers who were exposed to vibration (AOR = 0.419, *p*=0.021). Workers who were not exposed to rays/welding sparks were less likely to face occupational injury compared with workers who were exposed to rays (AOR = 0.366, *p*=0.012). Furthermore, workers who were not exposed to labor-intensive work had lower odds of occupational injury compared with workers who were exposed to labor-intensive work (AOR = 0.343, *p*=0.003) (Table 3).

## 4. Discussion

The one-year and two-week occupational injury prevalence was 42.7% (128) and 6.7% (20), respectively. This result agreed with the findings from Ayka Addis textile factory regarding the one-year and two-week prevalence which were 40.8% and 9.4%, respectively [9]. However, it was higher than that of the workers in Arba Minch textile factory (31.4%) [7], textile factories in the Amhara region (33.3%) [10], and Kombolcha textile factory (36.9%) [8].

In the present study, the two most injured body parts were hands (38 = 12.7%) and eyes (23 = 7.7%). Hand injuries may be caused by improper use of hand tools, not wearing gloves, inattention while handling sharp objects, and not following safety rules and guidelines [28], and eye injuries may be caused due to environmental conditions particularly dust particles (cotton) [29]. Hands and eyes are among the list of commonly affected body parts listed by the Ministry of Health Ethiopia [10]. In the same way, hands were among the most frequently injured body parts among workers from Arba Minch textile factory (39.7%) [7] and Ayka Addis textile factory (21.8%) [9] even though the prevalence of hand injuries in the present study was much lower than that in the mentioned studies. These differences may be due to differences in safety awareness, safety training, machine guards, working conditions, behavioral differences, provisions of PPEs, and nature of industries.

Monday showed the highest proportion of accidents (4.7%) and Sunday the least (1.0%). This may be because workers fraudulently claim that off-the-job weekend sprains and strains occurred at work on Monday [30], and a small proportion of workers are working as part-timers on Sunday. Similar findings were seen from the study conducted in Arba Minch textile factory: highest on Monday (21.3%) and lowest on Sunday (11%) [7].

It was observed that injuries were more common during the morning (7:00 AM–12:00 AM) (10.3%), afternoon (1:00 PM–6:00 PM) (9.3%), and after midnight (1:00 AM–6:00 AM) (8.7%). However, the least cases were reported during the evening shift (7:00 PM–12:00 PM) (4.0%). This may be due to lack of focus due to shortened sleep in the morning, rushing to go home in the afternoon, and shortened sleep after midnight [31]. The trend of injury time was in line with that of the workers from the Arba Minch textile factory [7], except that the prevalence in the present study was lower.

The bivariate regression analysis showed statistically significant differences (*p* < 0.05) in accidents due to differences in gender, job category, exposure to noise, exposure to vibration, exposure to rays/welding sparks, exposure to nonmoderate light, exposure to nonmoderate temperature, exposure to a dusty work area, prolonged work by standing in a limited area, exposure to biological hazards, and labor-intensive work. The males were 2.38-fold (1/0.421) more injured than females (COR = 0.421; 95% CI: 0.253–0.700). Workers from the weaving section (COR = 4.556; 95% CI: 1.934–10.730) were 10.45 (4.556/0.439), 7.52 (4.556/0.606), 5.12 (4.556/0.889), and 4.56 (4.556/1) times more injured than the administrative (COR = 0.436; 95% CI: 0.198–0.958), finishing (COR = 0.606; 95% CI: 0.279–1.315), engineering (COR = 0.889; 95% CI: 0.419–1.887), and spinning (ref) staff, respectively.

From the multiple regression analysis, statistically significant differences in injuries (*p* < 0.05) were observed due to variations in gender, job category, exposure to vibration, exposure to rays/welding sparks, and labor-intensive work. The males were 3.14 (1/0.318) times more injured compared with females (AOR = 0.318; 95% CI: 0.162–0.626). This finding was in line with the findings of the studies conducted in textile factories of the Amhara region [10] and textile factories in Northwest Ethiopia [11]. The difference in the levels of injury might be explained by the fact that female workers are usually assigned in less hazardous sections in textile industries and had different work schedules (the males worked relatively longer hours per day) [11, 32].

Most injuries occurred in the weaving section. The weaving section (AOR = 4.497; 95% CI: 1.557–12.990) resulted in injuries close to 8.37 (4.497/0.537) times higher than the finishing staff section (AOR = 0.537; 95% CI: 0.184–1.569), 4.497 (4.497/1) times higher than the spinning (ref) staff section, 3.92 (4.497/1.147) times higher than the engineering staff section (AOR = 1.147; 95% CI: 0.410–3.215), and 3.06 (4.497/1.471) times higher than the administrative staff section (AOR = 1.471; 95% CI: 0.445–4.868). However, it was different from that of Arba Minch [7] and Kombolcha [12] textile factories where such differences were not observed.

Study participants who were exposed to vibration were 2.39 (1/0.419) times more likely to be injured compared with workers who were not exposed to vibration (AOR = 0.419; 95% CI: 0.199–0.878), workers who were exposed to rays were 2.73 (1/0.366) times more likely to be injured compared with workers who were not exposed to rays (AOR = 0.366; 95% CI: 0.167–0.799), and workers who were exposed to labor-intensive work were 2.9 (1/0.343) times more likely to be injured compared with workers who were not exposed to labor-intensive work (AOR = 0.343; 95% CI: 0.167–0.703). Reports show that occupational exposure to vibration increases the risk of musculoskeletal pain in the back, neck, hands, shoulders, and hips [33], eye injury is associated with welding rays [34], and labor-intensive work is associated with occupational injuries [35].

## 5. Conclusions

The one-year and the two-week occupational injuries prevalence were 42.7% and 6.7%, respectively. Abrasion and eye injury were the two top injuries. Hands and eyes were the top injured body parts. Machines and falling/slipping caused most injuries. Statistically significant differences in injuries were observed due to variations in gender, job category, exposure to vibration, exposure to rays/welding sparks, and labor-intensive work. The weaving section was the risk factor significantly associated with occupational injuries.

## 6. Recommendations

Establishment of occupational safety and health committees; conducting occupational safety and health training; provision of quality personal protective equipment; conducting periodic occupational and safety supervisions; shifting workers on labor-intensive works; recording and analysis of injuries; enforcement of existing laws, policies, regulations, directives, and workplace standards in the country regarding occupational safety, health, and work environment conditions are recommended.

### 6.1. Limitations

The one-year injury prevalence was conducted using questionnaires. This might lead to recall bias. The recall biases may underestimate the injury prevalence. So, the findings from this study can be generalized to the whole worker population of Bahir Dar Textile SC.

### 6.2. Relevance of the Findings to Public Health

The findings of this study will serve as key for intervention against occupational injuries in Bahir Dar Textile SC in particular and Ethiopia in general. It will also play a role in minimizing the psychological, behavioral, social, vocational, and economic problems of the workers.

## Figures and Tables

**Table 1:** Sociodemographic characteristics of the respondents.

Variables	Number	Percent
Gender		
Male	195	65.0
Female	105	35.0

Age		
22–29	208	69.3
30–34	70	23.3
>34	22	7.3

Religion		
Orthodox	293	97.7
Muslim	6	2.0
Protestant	1	0.3

Ethnicity		
Amharas	297	99.0
Tigrayans	2	0.7
Oromo	1	0.3

Marital status		
Married	159	53.0
Single	136	45.3
Divorced	5	1.7

Education level		
No education	18	6.0
Primary	13	4.3
Secondary	49	16.3
Higher	220	73.3

Residence		
Urban	296	98.7
Rural	4	1.3

Job category		
Spinning	49	16.3
Weaving	53	17.7
Engineering	65	21.7
Finishing	64	21.3
Administrative staff	69	23.0

Employment status		
Permanent	296	98.7
Contract	4	1.3

Work experience		
<5 years	158	52.7
≥5 years	142	47.3

Monthly salary		
<2458 birr (<72.00 USD)	41	13.7
2459–3941 birr (72.03–115.45 USD)	108	36.0
3942–5776 birr (115.48–169.20 USD)	98	32.7
>5776 birr (>169.20 USD)	53	17.7

**Table 2:** Prevalence of occupational injuries among workers of Bahir Dar Textile SC, Ethiopia, April 2019.

Variables	Number	Percent
Injury in one year		
Yes	128	42.7
No	172	57.3

Injury in two weeks		
Yes	20	6.7
No	280	93.3

Frequency of occurrence injury in one year		
Once	59	19.7
More than once	69	23.0

Frequency of occurrence of injury in two weeks		
Once	18	6.0
More than once	2	0.7

Body part injured (128)		
Eye	23	7.7
Ear	16	5.3
Arm	9	3.0
Hand	38	12.7
Head	1	0.3
Upper leg	1	0.3
Lower leg	1	0.3
Tooth	1	0.3
Anterior trunk	1	0.3
Back	12	4.0
Knee	6	2.0
Toes	14	4.7
Face	2	0.7
Mixed	1	0.3
Others	2	0.7

Type of injury (*n* = 128)		
Abrasion	32	10.7
Burn	12	4.0
Cuts	8	2.7
Puncture	8	2.7
Sprain	16	5.3
Fracture	9	3.0
Dislocation	5	1.7
Eye injury	23	7.7
Ear injury	9	3.0
Suffocation	4	1.3
Other	2	0.7

Causes of injury (*n* = 128)		
Machines	68	22.7
Electricity	3	1.0
Hand tools	6	2.0
Explosive	2	0.7
Acids	5	1.7
Falling and slipping	19	6.3
Splinter	4	1.3
Collision	7	2.3
Mishandling	2	0.7
Falling objects	4	1.3
Improper usage	3	1.0
Others	5	1.7

Reasons for the accident (*n* = 128)		
Lack PPEs	59	19.7
Misuse of PPEs	17	5.7
Disorder of normal operation	8	2.7
Lack of safety training	18	6.0
Improper hand working instrument	13	4.3
Absence/inadequate machine safeguards	13	4.3

Day of injury		
Monday	14	4.7
Tuesday	9	3.0
Wednesday	7	2.3
Thursday	11	3.7
Friday	12	4.0
Saturday	4	1.3
Sunday	3	1.0
Do not remember	68	22.7

Time of injury (*n* = 128)		
In the morning (7:00 AM–12:00 AM)	28	9.3
In the afternoon (1:00 PM–6:00 PM)	31	10.3
In the evening (7:00 PM–12:00 PM)	12	4.0
After midnight (1:00 AM–6:00 AM)	26	8.7
Do not remember	31	10.3

Hospitalization (*n* = 300)		
Yes	84	28.0
No	216	72.0

Days lost due to injury (*n* = 300)		
2–6	39	13.0
7–14	24	8.0
15–30	18	6.0
>30	3	1.0

**Table 3:** Risk factors and their associations with the prevalence of occupational injuries.

Variables	Full model		Reduced model	
	COR (95% CI)	*p* value	AOR (95% CI)	*p* value
Gender		0.001		
Male	1.00		1.00	
Female	0.421 (0.253–0.700)	0.001^*∗*^	0.318 (0.162–0.626)	0.001^*∗*^

Age		0.159		
22–29	1.00		1.00	
30–34	0.841 (0.485–1.458)	0.537	0.773 (0.340–1.754)	0.537
>34	0.371 (0.132–1.043)	0.060	0.25 (0.054–1.152)	0.075

Marital status		0.329		
Married	1.00		—	—
Single	1.424 (0.894–2.267)	0.136	—	—
Divorced	1.10 (0.179–6.773)	0.918	—	—

Education level			—	—
No education	1.00		—	—
Primary	0.364 (0.06–2.194)	0.270	—	—
Secondary	1.379 (0.44–4.285)	0.578	—	—
Higher	1.577 (0.571–4.357)	0.379	—	—

Residence		0.735	—	—
Urban	1.00		—	—
Rural	1.407 (0.195–10.121)	0.735	—	—

Job category		0.000		0.003
Spinning	1.00		1.00	
Weaving	4.556 (1.934–10.730)	0.001^*∗*^	4.497 (1.557–12.990)	0.005^*∗*^
Engineering	0.889 (0.419–1.887)	0.759	1.147 (0.410–3.215)	0.794
Finishing	0.606 (0.279–1.315)	0.205	0.537 (0.184–1.569)	0.256
Administrative staff	0.436 (0.198–0.958)	0.039	1.471 (0.445–4.868)	0.527

Employment status		0.506	—	—
Permanent	1.00		—	—
Contract	0.462 (0.468–4.497)	0.506	—	—

Work experience		0.226		
<5 years	1.00		1.00	
≥5 years	0.752 (0.474–1.193)	0.226	1.061 (0.550–2.046)	0.861

Monthly salary		0.256	—	—
<2458 birr (<72.00 USD)	1.00		—	—
2459–3941 birr (72.03–115.45 USD)	1.789 (0.37–3.821)	0.133	—	—
3942–5776 birr (115.48–169.20 USD)	1.306 (0.602–2.834)	0.499	—	—
>5776 birr (>169.20 USD)	2.074 (0.886–4.853)	0.093	—	—

Existence of high noise or blare				
Yes	1.00		1.00	
No	0.236 (0.142–0.391)	0.000^*∗*^	0.766 (0.349–1.682)	0.507

Exposure to vibration				
Yes	1.00		1.00	
No	0.224 (0.135–0.373)	0.000^*∗*^	0.419 (0.199–0.878)	0.021^*∗*^

Exposure to rays/welding sparks				
Yes	1.00		1.00	
No	0.250 (0.149–0.421)	0.000^*∗*^	0.366 (0.167–0.799)	0.012^*∗*^

Exposure to nonmoderate light				
Yes	1.00		1.00	
No	0.439 (0.270–0.715)	0.001^*∗*^	1.303 (0.582–2.921)	0.520

Exposure to nonmoderate temperature				
Yes	1.00		1.00	
No	0.432 (0.269–0.693)	0.001^*∗*^	1.189 (0.568–2.490)	0.646

Exposure to chemical hazards				
Yes	1.00			
No	0.663 (0.390–1.127)	0.129	0.968 (0.446–2.103)	0.935

Exposure to dusty work area				
Yes	1.00			
No	0.412 (0.252–0.672)	0.000^*∗*^	0.937 (0.409–2.148)	0.878

Prolonged work by standing in a limited area				
Yes	1.00		1.00	
No	0.511 (0.305–0.857)	0.011^*∗*^	1.039 (0.515–2.099)	0.914

Exposure to biological hazards				
Yes	1.00			
No	0.531 (0.326–0.863)	0.011^*∗*^	0.615 (0.328–1.152)	0.129

Labor-intensive work				
Yes	1.00		1.00	
No	0.305 (0.182–0.513)	0.000^*∗*^	0.343 (0.167–0.703)	0.003^*∗*^

Work supervision		0.148		
Yes	1.00		1.00	
No	0.458 (0.159–1.320)	0.148	0.335 (0.077–1.450)	0.143

Alcohol consumption		0.422	—	—
Yes	1.00		—	—
No	0.761 (0.390–1.484)	0.422	—	—

Khat chewing		0.588	—	—
Yes	1.00		—	—
No	0.706 (0.2–2.492)	0.588	—	—

Cigarette smoking		0.506	—	—
Yes	1.00		—	—
No	2.163 (0.222–21.038)	0.506	—	—

Sleeping disturbance		0.061		
Yes	1.00		1.00	
No	0.643 (0.406–1.021)	0.061	0.759 (0.404–1.426)	0.392

Job satisfaction		0.082		
Yes	1.00		1.00	
No	1.561 (0.944–2.581)	0.082	0.902 (0.477–1.706)	0.751

PPE		0.607	—	—
Yes	1.00		—	—
No	0.881 (0.554–1.427)	0.607	—	—

COR, crude odds ratio; AOR, adjusted odds ratio; CI, confidence interval. ^*∗*^Statistically significant difference (*p* < 0.05).

## Data Availability

The datasets from which the conclusion was taken are available in the form of Microsoft Excel and are available on request.

## Abbreviations

AOR: Adjusted odds ratio
CI: Confidence interval
COR: Crude odds ratio
GDP: Gross domestic product
ILO: International labor organization
PPE: Personal protective equipment
SC: Share company.
